# Brainstem Involvement in Amyotrophic Lateral Sclerosis: A Combined Structural and Diffusion Tensor MRI Analysis

**DOI:** 10.3389/fnins.2021.675444

**Published:** 2021-06-02

**Authors:** Haining Li, Qiuli Zhang, Qianqian Duan, Jiaoting Jin, Fangfang Hu, Jingxia Dang, Ming Zhang

**Affiliations:** ^1^Department of Medical Imaging, The First Affiliated Hospital of Xi’an Jiaotong University, Xi’an, China; ^2^Department of Neurology, The First Affiliated Hospital of Xi’an Jiaotong University, Xi’an, China

**Keywords:** amyotrophic lateral sclerosis, magnetic resonance imaging, diffusion tensor imaging, shape analysis, brainstem

## Abstract

**Introduction:**

The brainstem is an important component in the pathology of amyotrophic lateral sclerosis (ALS). Although neuroimaging studies have shown multiple structural changes in ALS patients, few studies have investigated structural alterations in the brainstem. Herein, we compared the brainstem structure between patients with ALS and healthy controls.

**Methods:**

A total of 33 patients with ALS and 33 healthy controls were recruited in this study. T1-weighted and diffusion tensor imaging (DTI) were acquired on a 3 Tesla magnetic resonance imaging (3T MRI) scanner. Volumetric and vertex-wised approaches were implemented to assess the differences in the brainstem’s morphological features between the two groups. An atlas-based region of interest (ROI) analysis was performed to compare the white matter integrity of the brainstem between the two groups. Additionally, a correlation analysis was used to evaluate the relationship between ALS clinical characteristics and structural features.

**Results:**

Volumetric analyses showed no significant difference in the subregion volume of the brainstem between ALS patients and healthy controls. In the shape analyses, ALS patients had a local abnormal surface contraction in the ventral medulla oblongata and ventral pons. Compared with healthy controls, ALS patients showed significantly lower fractional anisotropy (FA) in the left corticospinal tract (CST) and bilateral frontopontine tracts (FPT) at the brainstem level, and higher radial diffusivity (RD) in bilateral CST and left FPT at the brainstem level by ROI analysis in DTI. Correlation analysis showed that disease severity was positively associated with FA in left CST and left FPT.

**Conclusion:**

These findings suggest that the brainstem in ALS suffers atrophy, and degenerative processes in the brainstem may reflect disease severity in ALS. These findings may be helpful for further understanding of potential neural mechanisms in ALS.

## Introduction

Amyotrophic lateral sclerosis (ALS) is a fatal neurodegenerative disease. It predominantly affects the upper and the lower motor neurons in the cerebral cortex, brainstem, and spinal cord, leading to progressive limb strength loss, dysarthria, drooling, tongue wasting, and dysphagia (Brown and Al-Chalabi, 2017; van Es et al., 2017). More recently, it appears that ALS pathology involves more than the motor system and pathological TAR DNA-binding protein 43 (TDP-43) inclusions have been confirmed to be in four stages in the whole brain, inclusive of extra-motor cortical and subcortical structures and the brainstem (Braak et al., 2013; Brettschneider et al., 2013; Qiao et al., 2020).

Advanced magnetic resonance imaging (MRI) methods are robust imaging techniques that enable the evaluation of neurological systems degeneration in ALS *in vivo* (Chio et al., 2014). Neuroimaging studies have revealed that anatomical and functional changes not only involve precentral gyrus (Walhout et al., 2015; Alshikho et al., 2018; Grapperon et al., 2019; Contarino et al., 2020) and corticospinal tract (CST) (Senda et al., 2017; Gorges et al., 2018; Ishaque et al., 2018; Broad et al., 2019; Baek et al., 2020) but also spread to the frontal cortex (Consonni et al., 2018, 2019), thalamus (Schönecker et al., 2018; Tu et al., 2018), and basal ganglia (Bede et al., 2013; Machts et al., 2015). On the other hand, quantitative volumetric studies in ALS have detected both gray and white atrophy results in global spinal cord atrophy in ALS (Rasoanandrianina et al., 2017; Paquin et al., 2018). Furthermore, CST and anterior horns degeneration and alterations in ALS are associated with functional impairment (Cohen-Adad et al., 2013). The brainstem pathology is considered as the “first stage” in the suggested four-stage pathological staging system based on pathological TDP-43 burden patterns (Brettschneider et al., 2013). However, few neuroimaging studies have addressed morphological changes in the brainstem and its subregions in ALS patients.

Corticospinal disruption is generally studied in ALS (Chio et al., 2014), while alterations in other sensorimotor pathways are less well understood. This could be because several experiments have studied morphological changes in the cerebrum, in which most of the ascending and descending brain pathways overlap. Besides, the sensorimotor pathways are separated in the brainstem (Vanderah and Gould, 2015; Gray, 2016). The brainstem is a fundamental structure that communicates motor and sensory information between the cerebral cortex and the spinal cord. Previous researches using diffusion tensor imaging (DTI) have confirmed the extensive degeneration of the brain in ALS patients. However, most studies only established the most significant alterations in CST in the ALS brainstem areas, with limited research investigating extra significant white matter tract alterations in the brainstem regions in the ALS group.

Therefore, the current study aimed to reveal the patterns of focal gray matter atrophy and white matter damage in brainstem regions of ALS patients. Both volumetric and vertex-wise approaches were performed for the brainstem to compare ALS patients and healthy controls. Then the region of interest (ROI) analysis was used to compare diffusion metrics in ALS patients with the white matter in healthy controls. Additionally, correlation analysis was used to evaluate the relationship between ALS clinical features and volume or diffusion metrics.

## Materials and Methods

### Participants

Thirty-three patients were recruited. Twenty-two patients with definite ALS and 11 patients with probable ALS were clinically diagnosed based on the revised El Escorial criteria. None of the ALS patients in this study had a family history of ALS. With respect to ALS phenotypes classification, as formerly identified by Chiò et al. (2011), 29 patients displayed classic phenotype and four exhibited bulbar phenotype. To evaluate disease severity, the Revised ALS Functional Rating Scale (ALSFRS-R) (Brooks et al., 2000) was used. The duration of the disease was calculated from the onset of symptoms to the acquisition of MR imaging in months. To modify the degree of disability for disease duration, the rate of disease progression was calculated using the following formula: (48-ALSFRS-R score)/(disease duration). None of the ALS patients had a history of cerebrovascular events, intracranial pathology, or other neurological diseases. None of the ALS patients had clinical signs of frontotemporal dementia. Mini-Mental Status Examination (MMSE) was used to assess general cognitive functions. Thirty-three right-handed healthy controls matched for age and gender were recruited. There was no history of mental and neurological problems in healthy controls.

Written informed consent was obtained from all participants. Ethical approval for all procedures was obtained from the Ethics Committee of the First Affiliated Hospital of Xi’an Jiaotong University in advance.

### MRI Acquisition

Magnetic resonance imaging data were acquired on a 3 Tesla GE scanners (General Electric Healthcare, Milwaukee, WI, United States) using an eight-channel parallel head coil. The high-resolution T1-weighted MRI images of the brain were obtained using a 3D T1 fast spoiled gradient-echo sequence with the following parameters: TE = 4.8 ms; TR = 10.8 ms; field of view (FOV) = 256 mm × 256 mm; matrix = 256 × 256; voxel size = 1 × 1 × 1 mm. The conventional T2 weighted imaging, fluid-attenuated inversion recovery (FLAIR) sequences were obtained to rule out cerebral infarction, tumors, and other incidental findings. Whole-brain DTI images were performed using an echo-planar imaging sequence with the following parameters: TR = 14 s, TE = 90.7 ms, FOV = 256 mm × 256 mm, matrix = 128 × 128, slice thickness = 2.5 mm, 35 isotropic directions, *b*-value = 1000 s/mm^1^. In addition, one scan without diffusion weighting (*b* = 0 s/mm^2^) was acquired. Resting-state functional MRI (reported elsewhere) was also obtained. The total acquisition time was approximately 30 min for each subject. During scanning, a tight but comfortable sponge pad inside the head coil was used to restrict head motion.

### Image Analysis

FreeSurfer software version 6.0 (Massachusetts General Hospital, Boston, MA, United States^2^) was used for the preprocessing of T1-weighted images (Fischl et al., 1999). The main recon stream (“recon-all”) in FreeSurfer is used for volumetric segmentation, specifically including motion correction, skull-stripping, non-parametric non-uniform intensity normalization, Talairach transformation (affine transform from the original volume to the MNI305 atlas), volumetric registration, and topology correction (Fischl et al., 2001; Segonne et al., 2007; Reuter et al., 2010). Automated segmentation and volume computations of the whole brainstem and four brainstem substructures [pons, midbrain, medulla, and superior cerebellar peduncle (SCP)] were completed using the brainstem substructures toolbox implemented in FreeSurfer software. Segmentation was conducted using a robust and accurate Bayesian algorithm relying on a probabilistic atlas of the brainstem and neighboring anatomical structures (Iglesias et al., 2015). Each subject’s T1 imaging outputs in all processes were carefully inspected for errors by two trained independent researchers to ensure the quality of brainstem segmentation.

Moreover, the total intracranial volume (TIV) was calculated for each participant with SIENAX (Smith et al., 2002) in the FMRIB Software Library (FSL version 6.0.3^2^) (Jenkinson et al., 2012), which was used as a covariate for subsequent volumetric comparisons.

While volumetric analysis can only offer information about the total size of the brainstem, vertex-wise subcortical shape analysis can identify changes in the shape of the brainstem and offer more information about regional abnormalities in the brainstem. Vertex-wise shape analysis was achieved through the algorithm FIRST, (Patenaude et al., 2011), a model-based segmentation and registration module implemented in FSL software. This method is based on a Bayesian framework model; the multivariate Gaussian shape and appearance of subcortical structures are constructed from a large set of manually labeled images (336 brains) provided by the Center for Morphometric Analysis, Massachusetts General Hospital, Boston. During registration, raw 3D T1 images are transformed to MNI152 template by standard 12 degrees of freedom and accurately registered to a Montreal Neurological Institute (MNI) 152 brainstem mask to exclude voxels outside the brainstem region. Then brainstem was automatically segmented based on a Bayesian framework. Afterward, the surface mesh output of the brainstem, which was used for surface-based vertex analyses, was generated.

Diffusion tensor imaging data processing and analysis were performed using software tools from FSL. Eddy current induced distortions and head motion in the diffusion-weighted images was corrected using EDDY tools provided in the FSL, (Andersson and Sotiropoulos, 2016). Then the quality of the dataset was assessed using QUAD and SQUAD (automated EDDY quality control framework in FSL), (Bastiani et al., 2019). The quality control criteria were set as average absolute volume to volume head motion of <3 mm or total outliers <5% by referencing the previous literature, (Zheng et al., 2021). An example of a quality control report is added in Supplementary Material. Skull stripping was performed for each participant using FSL’s Brain Extraction Tool (BET), (Smith, 2002). Afterward, by fitting a tensor model to the raw diffusion data, quantitative DTI parameters of fractional anisotropy (FA), axial diffusivity (AD), mean diffusivity (MD), and radial diffusivity (RD) images were calculated.

All subjects’ FA, MD, AD, and RD images were then registered to the standard MNI152 space using a non-linear registration algorithm (FSL’s FLIRT and FNIRT) (Smith et al., 2004; Greve and Fischl, 2009), which uses a b-spline representation of the registration warp field (Rueckert et al., 1999).

**FIGURE 1:**
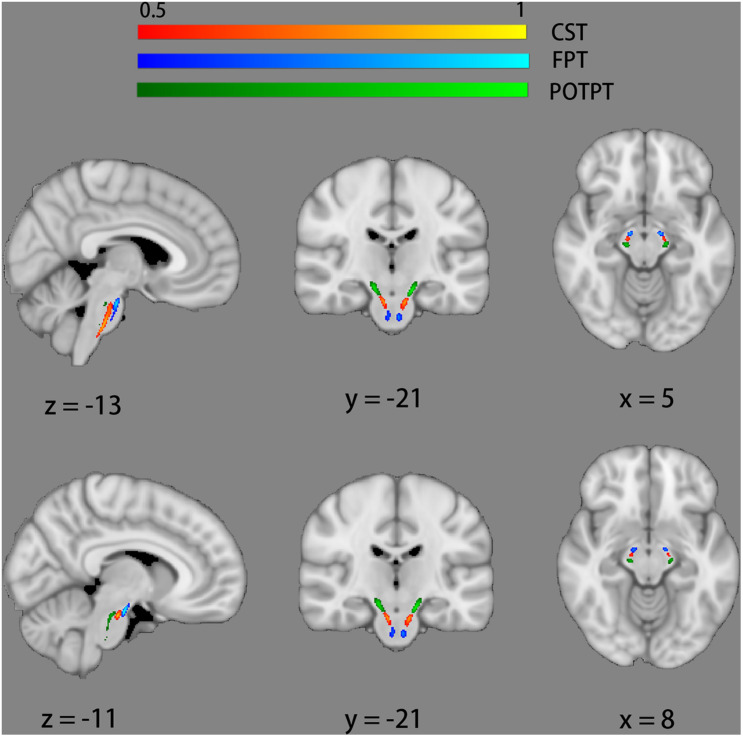
Six regions of interest of motor tracts defined by probabilistic atlases of 23 brainstem bundles. The bilateral corticospinal tracts (CST) are marked red-yellow. The bilateral fronto-pontine tracts (FPT) are displayed in blue. The bilateral parieto-occipito-temporo-pontine tracts (POTPT) are represented as green.

An atlas-based ROI analysis was performed to compare diffusion metrics of brainstem fiber pathways in ALS patients with healthy controls. Recently, a novel probabilistic atlas of 23 brainstem pathways using the Human Connectome Project (HCP) data was developed and publicly distributed (Tang et al., 2018). The atlas was in the MNI152 space, which can be downloaded on NITRIC^3^. ROIs were defined by anatomic marks obtained from the 23 brainstem pathways atlas and six ROIs of motor tracts were identified, which were respectively bilateral CST, bilateral frontopontine tracts (FPT), and bilateral parieto-occipito-temporo-pontine tracts (POTPT) (Figure 1). The average values within each region were calculated.

### Statistics Analysis

Normal distribution assumption was examined by Kolmogorov–Smirnov tests. Group differences in the clinical data were evaluated using a *t*-test for continuous variables and Chi-square tests for discrete variables. All non-voxel-wise statistical analyses were carried out with Statistical Package for the Social Sciences (SPSS) V.20 (IBM SPSS, IBM Corp., NY, United States).

An analysis of covariance (ANCOVA) was used to test for volume differences of the whole brainstem and its subregions between groups, adjusting for the effect of age and TIV to avoid spurious results.

Meanwhile, ANCOVA was performed to test for changes in DTI parameters within the brainstem fiber pathways between groups with age as a covariate. The analysis was carried out with average values of FA, MD, RD, and AD, respectively. After Bonferroni correction, *P* < 0.008 (0.05/6) was considered statistically significant. In line with recent recommendations (Chen et al., 2017), partial eta squared was calculated to estimate effect sizes.

For statistical analysis of brainstem shape data, general linear models consisting of age as a covariate were used for permutation-based non-parametric statistics. The non-parametric permutation approach (5000 permutations) was accomplished by the randomize tool commanded in FSL, (Winkler et al., 2014). Results with *P* < 0.05 were taken into consideration significantly after threshold-free cluster enhancement (TFCE) and family-wise error corrected (FWE) correction for multiple comparisons (Smith and Nichols, 2009).

Due to skewed distributions, the Spearman correlation test was used to analyze the relationship between different variables. Correlation significant at *P* < 0.008 (0.05/6) was considered statistically significant after Bonferroni correction for multiple comparisons.

## Results

### Demographic and Clinical Characteristics

The demographic and clinical data of enrolled individuals are summarized in Table 1. There were no significant differences in age (*P* = 0.852) or gender (*P* = 0.622) between patient groups and the healthy controls. There were no significant differences in MMSE (*P* = 0.07).

**TABLE 1:** Demographic characteristics of the cohorts.

**Characteristics**	**ALS patients**	**Healthy controls**
Number of participants	33	33
Gender (male/female)	18/15	16/17
Age at MRI scan	52.39 (1.56)	51.97 (1.63)
Handedness (right/left)	33/0	33/0
Site of ALS onset		
Bulbar	4	N/A
Upper limb	21	N/A
Lower limb	8	N/A
Disease duration (months)	16.82 (13.42)	N/A
Disease progression rate	0.87 (0.91)	N/A
All ALSFRS-R score (/48)	38.61 (6.57)	N/A
ALSFRS-R bulbar subscore (/12)	10.86 (1.84)	N/A
ALSFRS-R upper limb subscore (/12)	7.12 (3.40)	N/A
ALSFRS-R lower limb subscore (/12)	8.76 (2.91)	N/A
ALSFRS-R respiration subscore (/12)	12 (0)	N/A
MMSE	27.91 (1.99)	28.61 (0.86)

*Values are given in mean (standard deviation) format where appropriate. ALSFRS-R, Revised Amyotrophic Lateral Sclerosis Functional Rating Scale. MMSE, Mini-Mental State Examination.*

### Comparisons of the Volume and Shape of Brainstem Structures Between ALS Patients and Healthy Controls

There were no significant differences in the volumes of the whole brainstem and three brainstem subregions between ALS patients and the healthy control group (Table 2).

**TABLE 2:** Difference of volumes of brainstem regions between the ALS and healthy controls group.

**Brainstem region**	**ALS patients**	**Healthy controls**	***F***	**Partial eta squared**	***P*-value**
Medulla oblongata	4378.21 ± 451.64	4210.41 ± 437.02	2.352	0.037	0.130
Pons	12,947.33 ± 1950.17	13,149.50 ± 1440.01	0.522	0.008	0.473
Midbrain	5736.68 ± 734.86	5694.19 ± 576.41	0.887	0.014	0.701
SCP	244.52 ± 55.25	234.57 ± 40.55	0.149	0.002	0.350
Whole brainstem	23,305.89 ± 2994.61	23,289.52 ± 2114.33	0.001	<0.001	0.970

*Data are mean ± standard deviation (mm^3^). The *P*-values are obtained using analysis of covariance (ANCOVA) adjusted for age, gender, and total intracranial volume as covariates. SCP, superior cerebellar peduncle.*

**FIGURE 2:**
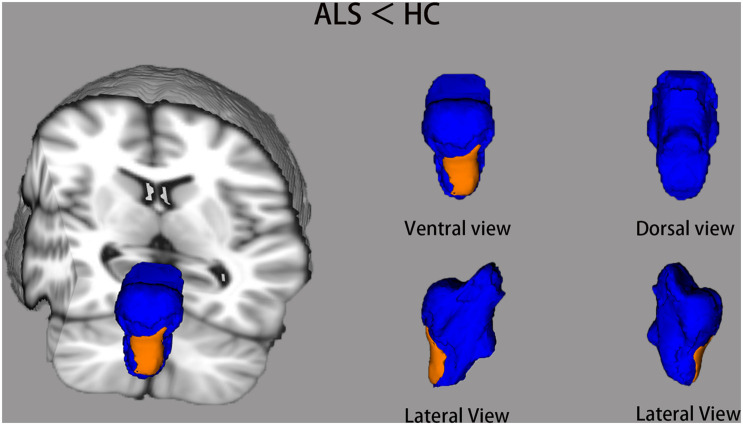
The brainstem shape differences between ALS groups and healthy controls (HC) using vertex-wise surface analyses. The blue color indicates the 3D brainstem mesh. The regions in orange represent ALS-related local shape deformations (ALS < HC). The results are corrected for multiple testing with the threshold-free cluster enhancement (TFCE) and family-wise error (FWE) method (*P* < 0.05).

Comparisons of the vertex-wise shape of brainstem structures between ALS patients and healthy controls are shown in Figure 2. Automated brainstem vertex-wise analysis revealed that the ventral medulla oblongata and a small part of the ventral pons had significant group differences in the ALS group than in the healthy controls group, following TFCE and FWE correction (*P* < 0.05).

### Comparison of DTI Findings With Atlas-Based ROI Analysis

**FIGURE 3:**
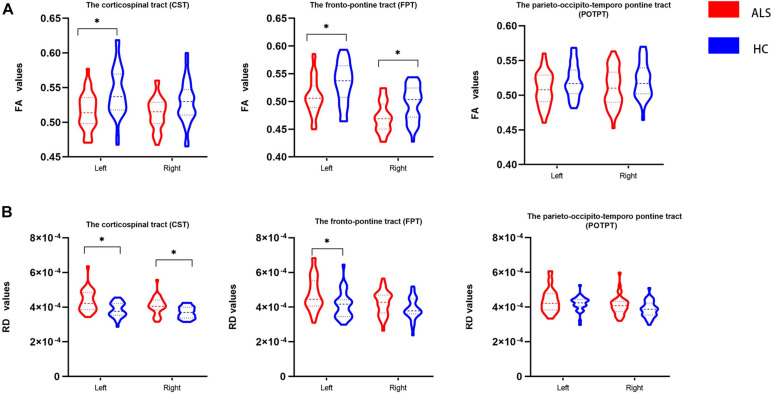
The violin plot depicts the diffusion measures from the region of interest analysis of ALS patients and healthy control groups. The blue color represents healthy controls (HC), and the red represents ALS patients. Inner violin plot shows the quartile, median. Asterisk indicates significance after Bonferroni correction. **(A)** Comparison of FA values between groups. **(B)** Comparison of RD values between groups.

Figure 3 and Table 3 show comparisons of diffusion metrics between ALS patients and healthy controls for six motor tracts of brainstem pathways. In the ROI analysis, ALS patients had significantly decreased FA values in the left CST but increased RD values in the bilateral CST at the brainstem level (*P* < 0.008). In addition, ALS patients had significantly decreased FA values in the bilateral FPT but increased RD values in the left FPT (*P* < 0.008).

**TABLE 3:** Group comparisons of the mean DTI parameters in ALS patients and healthy controls.

**Region**	**DTI parameters**	**ALS patients**	**Healthy controls**	***F***	**Partial eta squared**	***P*-value**
L-CST	FA	0.52 ± 0.026	0.54 ± 0.035	12.571	0.169	<0.001*
	MD	0.82 ± 0.075	0.80 ± 0.085	0.95	0.015	0.333
	AD	1.57 ± 0.113	1.61 ± 0.143	1.779	0.028	0.187
	RD	0.44 ± 0.063	0.38 ± 0.043	15.136	0.196	<0.001*
R-CST	FA	0.51 ± 0.023	0.53 ± 0.030	6.445	0.094	0.014
	MD	0.81 ± 0.082	0.80 ± 0.104	0.049	0.001	0.826
	AD	1.60 ± 0.109	1.65 ± 0.178	2.506	0.039	0.118
	RD	0.41 ± 0.050	0.37 ± 0.033	14.782	0.193	<0.001*
L-FPT	FA	0.51 ± 0.032	0.53 ± 0.037	8.647	0.122	0.005*
	MD	0.89 ± 0.129	0.86 ± 0.204	0.155	0.002	0.695
	AD	1.71 ± 0.187	1.73 ± 0.302	0.235	0.004	0.63
	RD	0.48 ± 0.096	0.41 ± 0.078	8.188	0.117	0.006*
R-FPT	FA	0.47 ± 0.027	0.50 ± 0.032	11.324	0.154	<0.001*
	MD	0.84 ± 0.129	0.83 ± 0.174	0.023	0.003	0.88
	AD	1.66 ± 0.171	1.70 ± 0.303	0.654	0.01	0.422
	RD	0.43 ± 0.070	0.39 ± 0.063	4.768	0.071	0.033
L-POTPT	FA	0.51 ± 0.024	0.52 ± 0.024	4.061	0.061	0.048
	MD	0.77 ± 0.064	0.77 ± 0.047	0.05	0.001	0.823
	AD	1.43 ± 0.092	1.46 ± 0.084	2.172	0.034	0.146
	RD	0.44 ± 0.069	0.42 ± 0.042	1.555	0.024	0.217
R-POTPT	FA	0.51 ± 0.028	0.53 ± 0.026	3.094	0.048	0.083
	MD	0.75 ± 0.054	0.75 ± 0.053	0.283	0.005	0.597
	AD	1.43 ± 0.090	1.48 ± 0.116	5.217	0.078	0.046
	RD	0.41 ± 0.057	0.39 ± 0.047	3.696	0.056	0.059

*All values are expressed as the mean ± standard deviation. Mean diffusivity (MD), axial diffusivity (AD), and radial diffusivity (RD) × 10^4^ mm^2^/s. Asterisk indicates significance after Bonferroni correction. P-values are presented before Bonferroni correction. L, left; R, right. CST, corticospinal tract. FPT, fronto-pontine tract. POTPT, parieto-occipito-temporo-pontine tract.*

### Correlations Between Imaging Findings and Clinical Characteristics

**FIGURE 4:**
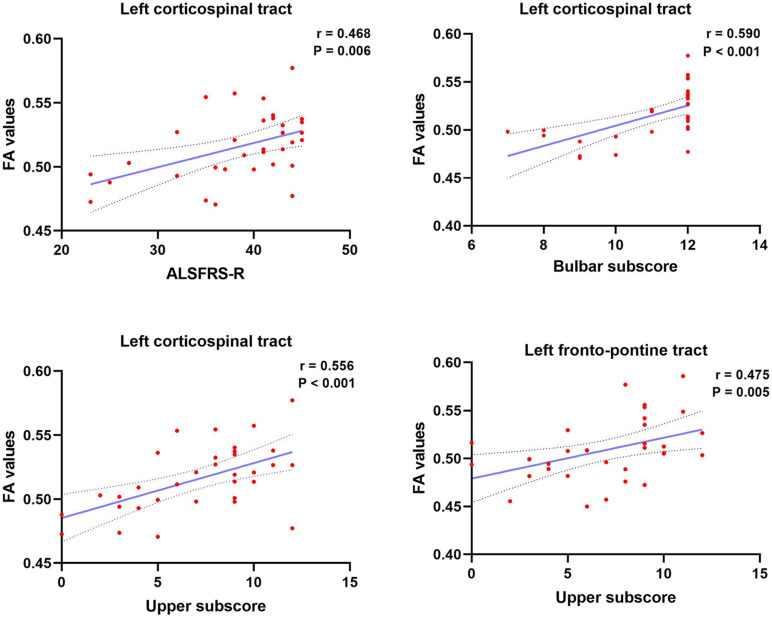
Significant clinical correlates of ALSFRS-R, ALSFRS-R bulbar subscore, and upper limb subscore, with FA values of brainstem pathways in patients with ALS. Dotted lines represent the 95% confidence region of the linear model fit. *P* < 0.008 (0.05/6) was considered statistically significant after Bonferroni correction. ALSFRS-R, Revised Amyotrophic Lateral Sclerosis Functional Rating Scale.

As shown in Figure 4, ALSFRS-R showed significant positive correlations with FA values of the left CST (*r* = 0.468, *P* = 0.006). Similarly, the bulbar sub-score showed positive correlations with FA values in the left CST (*r* = 0.590, *P* < 0.001). Moreover, the upper sub-score showed significant positive correlations with left CST (*r* = 0.550, *P* < 0.001) and the left FPT (*r* = 0.475, *P* = 0.005). There were no other significant associations between imaging findings (volumetric metrics and vertex data) and clinical data.

## Discussion

This study demonstrated the patterns of structural degeneration of the brainstem in ALS using the volumetric and surface-based approach, in addition to selective fiber integrity investigation. The results revealed that ALS patients encounter specific brainstem volume loss and white matter degeneration. The volume loss in the brainstem is mainly characterized by the local shape deformation in the medulla oblongata and pons. Selective degeneration in the CST and FPT related to clinical disease deterioration.

Our vertex-wised analyses identified shape deformations of the medulla oblongata in ALS patients compared with healthy controls. A fraction of involvement of posterior pons was also demonstrated in ALS patients. A previous study by Bede et al. (2019) proved significant alterations of the brainstem in ALS patients. Brainstem pathology refers to stage one of the lately proposed phosphorylated TDP-43 (pTDP-43) pathological staging scheme, which is characterized by the involvement in the brainstem motor nuclei of cranial V, VII, and X–XII of TDP-43 pathology (Braak et al., 2013; Brettschneider et al., 2013). Furthermore, the aforementioned cranial nerves are mainly distributed in the medulla oblongata and pons. Our volumetric analysis did not reveal the general volume reduction of the gray matter related to ALS patients in the three subregions of the brainstem. Metabolism has been shown to increase in bilateral midbrain and pons areas in ALS patients, suggesting the local activation of microglia and astrocytes in the brainstem of ALS patients (Haukedal and Freude, 2019; Volonté et al., 2019). Studies of ALS postmortem (Cardenas et al., 2017) and mouse model pathology (Chiarotto et al., 2019; Espejo-Porras et al., 2019) have shown astrogliosis and active microglia surrounding the neurodegeneration region. One possible reason for the negative result may be that the increased number of astrocytes in brain regions influenced by ALS occupies the place of the dead neurons, compensating for volume loss by shrinkage of neurons.

The relationship between the volume and the shape structure on the brainstem surface is yet to be understood (Rahayel et al., 2019). Volume analysis only provides information about the global size of the brainstem and neither presents any location information for volume changes nor offers any information about the shape (Patenaude et al., 2011). The present results showed that the estimated total brainstem volume might not capture structural alterations in ALS patients. The change in shape may precede volume changes of the brainstem. Therefore, a shape-based morphological analysis could be a useful tool for detecting early brainstem atrophy in ALS.

In our study, significantly reduced FA values in the left CST at the brainstem level and increased RD values in the bilateral CST at the brainstem level were observed. Other DTI studies have also investigated significant abnormalities at brainstem levels of the CST in ALS patients (Cardenas-Blanco et al., 2014; Floeter et al., 2014; Schuster et al., 2016; Baek et al., 2020). A previous multicenter imaging study detected a reduced FA value within CST at the brainstem level (Müller et al., 2016). Furthermore, a recent voxel-based meta-analysis revealed significant FA reduction in the right CST that stretched to the right cerebral peduncle (Zhang et al., 2018). Decreased FA and increased RD values commonly reflect some degree of impaired fiber integrity (Hutton et al., 2020). FA assesses the extent of anisotropy of diffusion processes (van Veluw et al., 2019). RD is a straight estimate that represents vertical directions to the tract, offering more indirect information on pathological changes in the axons or myelin compared to FA or MD (Fattah et al., 2020). Recent research has validated RD metrics as sensitive to characterize myelin (Geeraert et al., 2018). In addition, pathological changes of the white matter, including myelin damage and axonal degeneration, have been revealed in animal ALS models and postmortem studies of ALS patients (Kang et al., 2013; Wang et al., 2020). Therefore, the DTI results indicated a disruption and degeneration of CST in the brainstem region.

The present analysis showed a significant positive association between FA values of the left CST and total ALSFRS-R, which is consistent with former DTI studies (Bao et al., 2018; Kassubek et al., 2018). Lower performance in the bulbar function and the upper limb function was associated with lower FA values of the left CST in the brainstem level in ALS patients. A previous study has found an increase in functional brain network connectivity along with ALS disease progression (Sorrentino et al., 2018). In addition, a previous neuroimaging study showed that disease-related gray and white matter changes in ALS patients propagated from the motor to extra-motor areas as ALS progresses (Trojsi et al., 2015). The current findings may reinforce the notion that disease may propagate through axonal pathways in multiple regions of the brain. Following this evidence, we suggest that the CST at the brainstem level may be a reliable region for monitoring disease severity. Meanwhile, the results may provide further *in vivo* evidence for the proposed staging scheme of ALS-associated pathology (Braak et al., 2013; Brettschneider et al., 2013).

Patients with ALS had decreased FA values in bilateral FPT underlying the brainstem and increased RD values in the left FPT in the brainstem level. FPT is part of the cortico-ponto-cerebellar system, which is a white matter tract stretching from the cerebral cortex via pontine nuclei to the cerebellum (Jang et al., 2014; Palesi et al., 2017). This system plays an important role in movement regulation and modulates higher cognitive functions (Palesi et al., 2017; Wagner and Luo, 2020). Based on a multicenter DTI study, corticopontine tract (CPT) FA decreases have been related to stage 2 of a lately suggested pathological staging system (Kassubek et al., 2014; Müller and Kassubek, 2018). Moreover, a DTI study has shown that disruption of white matter integrity of the cortico-ponto-cerebellar system in ALS patients was associated with the pseudobulbar syndrome, (Floeter et al., 2014). Further, cortico-ponto-cerebellar system microstructural changes have been related to memory impairment in ALS patients, (Trojsi et al., 2020)., Mean FA of the CPT was reduced in ALS patients in a longitudinal multicenter study (Kalra et al., 2020). The CPT is associated with hand dominance-related alterations (Kim et al., 2019). In addition, previous researches have demonstrated that the integrity of the CPT in patients with chronic stroke affects the residual upper limb function (Schulz et al., 2015; Guder et al., 2020). A positive association was observed between the FA of the left FPT and the upper limb sub-score, which concurs with the association between the FA of the CPT and the finger-tapping-score in ALS patients (Kalra et al., 2020). Thus, the identified alterations may contribute to abnormal upper limb function of ALS patients.

However, some limitations should be acknowledged in our study. First, the spatial resolution of DTI in our study was relatively low, and there were still some residual distortions in the brainstem even after the application of correction procedures. These may reduce the interpretability of the results. Therefore, more high-resolution and accurate assessment methods, such as High Angular Resolution Diffusion-weighted Imaging or Diffusion Spectrum Imaging, need to be performed in future studies. Second, the sample size of our patients is relatively small. It is undoubtedly that the limited sample size of a single-center study is challenged by inherent disease heterogeneity in ALS. Therefore, multicenter cooperation is required to solve this problem. In addition, the limitation of this study lies in its cross-sectional nature. Thus, further studies should be performed to assess the changes of longitudinal volume and diffusion in the brainstem of ALS patients.

## Conclusion

In summary, the present study provides evidence for brainstem involvement in ALS patients, which is characterized by local atrophy in the medulla oblongata and white matter degeneration in the CST and FPT in the brainstem region. Furthermore, FA reduction in the left CST and FPT may reflect the severity of the disease. These findings may be helpful for further understanding of potential neural mechanisms in ALS.

## Data Availability Statement

The original contributions presented in the study are included in the article/Supplementary Material, further inquiries can be directed to the corresponding author/s.

## Ethics Statement

The studies involving human participants were reviewed and approved by the Ethics Committee of The First Affiliated Hospital of Xi’an Jiaotong University. The patients/participants provided their written informed consent to participate in this study.

## Author Contributions

MZ, HL, and QZ designed the experiments and wrote the manuscript. HL, QD, and QZ carried out the experiment. HL, JJ, and FH performed the acquisition of data. HL, JD, and QZ analyzed and interpreted the data. All authors read and approved the final manuscript.

## Conflict of Interest

The authors declare that the research was conducted in the absence of any commercial or financial relationships that could be construed as a potential conflict of interest.

## Footnotes

1 http://www.fmrib.ox.ac.uk/fsl

2 http://surfer.nmr.mgh.harvard.edu

3 https://www.nitrc.org/projects/brainstem_atlas/
