# Supplementary material for: Brainstem Involvement in Amyotrophic Lateral Sclerosis: A Combined Structural and Diffusion Tensor MRI Analysis
Source: Front Neurosci. 2021 Jun 2;15:675444. doi: 10.3389/fnins.2021.675444 (PMC8206526; doi:10.3389/fnins.2021.675444)
Supplement: Supplementary file 1 [file Data_Sheet_1.PDF]

Example of single subject quality control report

Single subject QC report generated using eddy quad v1.0.2

When using eddy and its QC tools, we ask you to please reference the papers describing the different aspects of the modelling and corrections. The following suggestion for a methods section and list of references has been tailored for you based on your eddy command line.

METHODS
Eddy current-induced distortions and gross subject movement were corrected using the "eddy" tool (Andersson & Sotiropoulos, 2016). The quality of the dataset was assessed using the eddy QC tools (Bastiani et al., 2019).

REFERENCES

Jesper L.R. Andersson and Stamatios N. Sotiropoulos. 2016. An integrated approach to correction for off-resonance effects and subject movement in diffusion MR imaging. NeuroImage 125:1063-1078

Matteo Bastiani, Michiel Cottaar, Sean P. Fitzgibbon, Sana Suri, Fidel Alfaro-Almagro, Stamatios N. Sotiropoulos, Saad Jbabdi and Jesper L.R. Andersson. 2019. Automated quality control for within and between studies diffusion MRI data using a non-parametric framework for movement and distortion correction. NeuroImage 184:801-812

Volume-to-volume motion

Average abs. motion (mm)	0.53
Average rel. motion (mm)	0.02
Average x translation (mm)	0.07
Average y translation (mm)	0.05
Average z translation (mm)	0.32
Average x rotation (deg)	-0.47
Average y rotation (deg)	-0.35
Average z rotation (deg)	-0.07

Outliers

Total outliers (%)	0.05
Outliers (b=1000 s/mm^2)	0.05
Outliers (PE dir=[0. 1. 0.])	0.05

Eddy currents

Std Dev EC linear term (x)	0.00
Std Dev EC linear term (y)	0.00
Std Dev EC linear term (z)	0.01

Average DW signal (b=0)

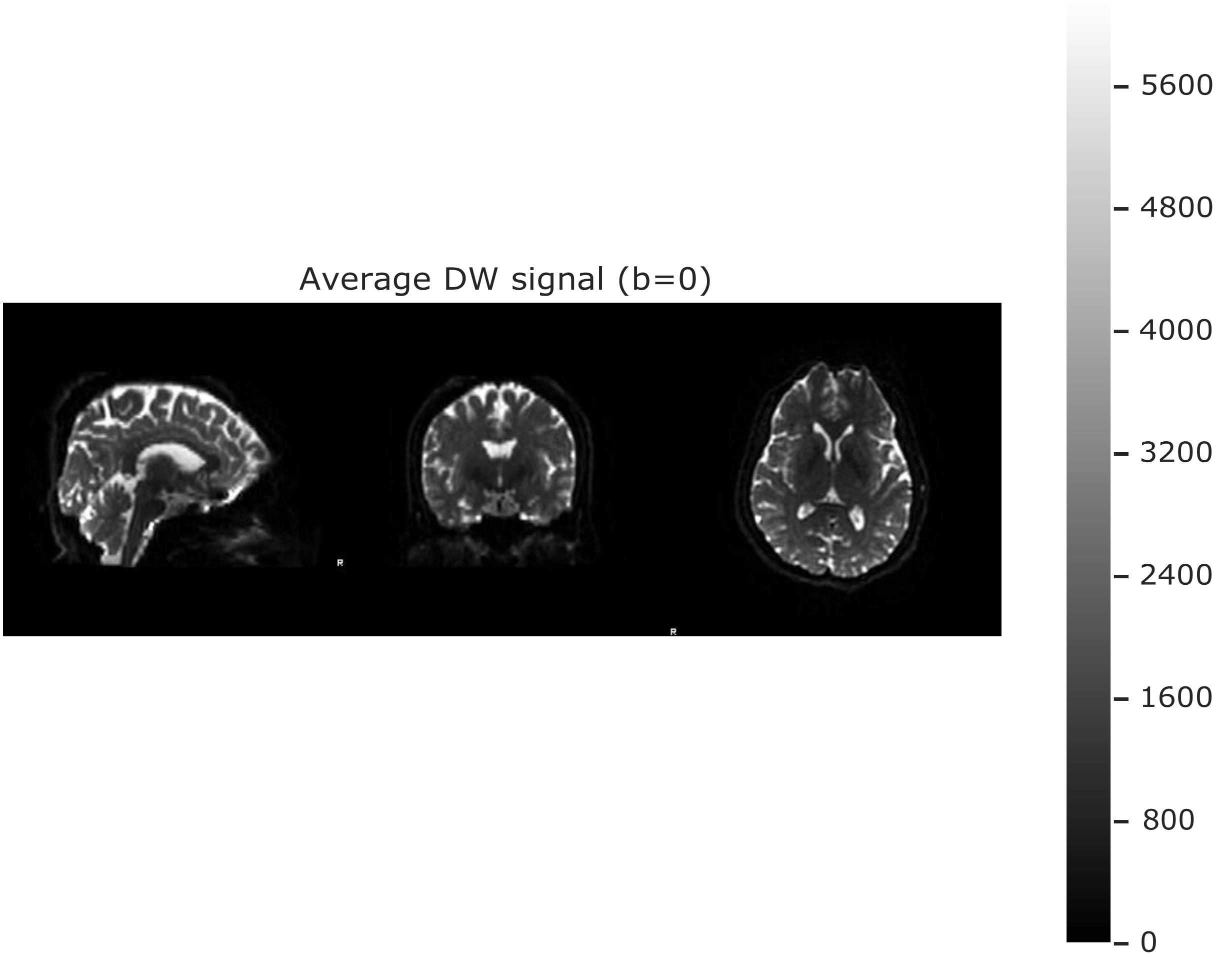

Average DW signal (b=1000)

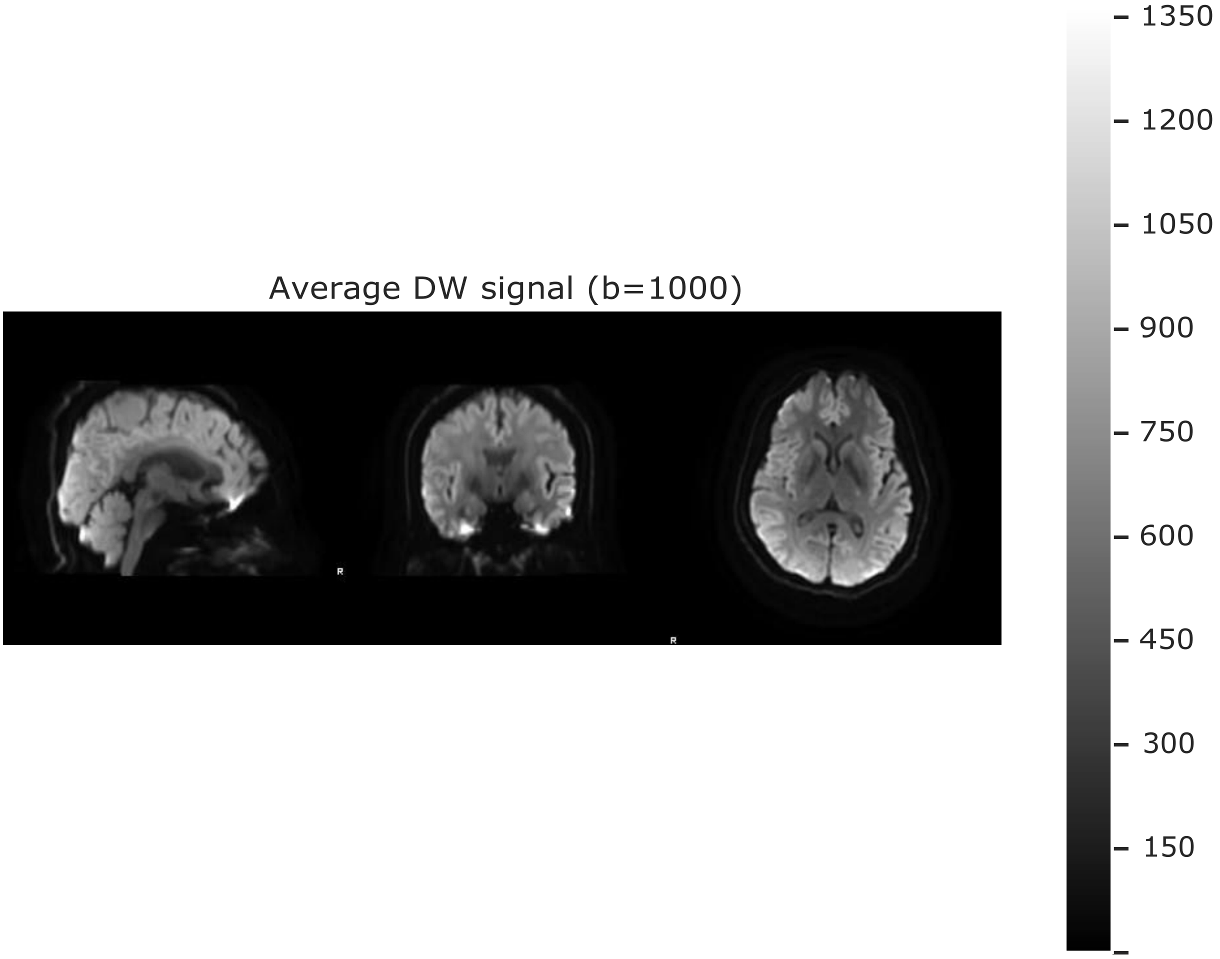

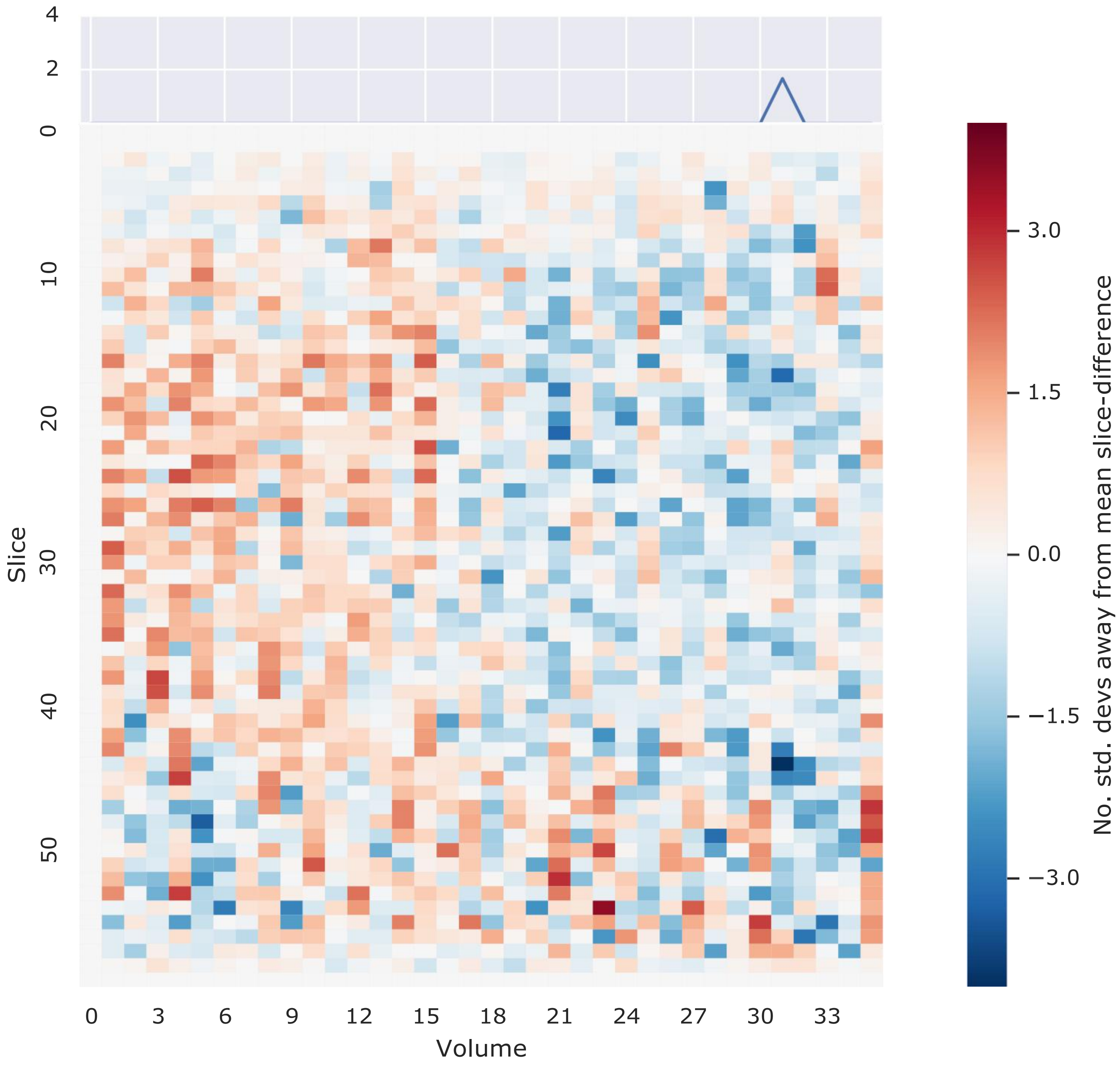

4
2
0
Slice
0
10
20
30
40
50
0
3
6
9
12
15
18
21
24
27
30
33
Volume
3.0
1.5
0.0
−1.5
−3.0
No. std. devs away from mean slice-difference

Example of group quality control report

Group QC report generated using eddy squad v1.0.2

When using eddy and its QC tools, we ask you to please reference the papers describing the different aspects of the modelling and corrections. The (Andersson & Sotiropoulos, 2016) paper is the main eddy reference and should always be be cited when using eddy. The (Bastiani et al., 2019) paper is the main eddy QC reference and should always be be cited when using the QC tools. When using topup to estimate a fieldmap prior to running eddy one should also cite (Andersson et al., 2003). If you have used eddy to detect and replace outlier slices (by adding --repol to the eddy command line), please also cite (Andersson et al., 2016). If eddy was used to estimate and correct for intra-volume (slice-to-vol) movement by specifying --mporder, please also cite (Andersson et al., 2017). Finally, if you asked eddy to model how the susceptibility-induced distortions change as a consequence of subject movement (with the --estimate_move_by_susceptibility flag), please cite (Andersson et al., 2018).

REFERENCES

Jesper L.R. Andersson, Stefan Skare and John Ashburner. 2003. How to correct susceptibility distortions in spin-echo echo-planar images: application to diffusion tensor imaging. NeuroImage 20:870-888

Jesper L.R. Andersson and Stamatios N. Sotiropoulos. 2016. An integrated approach to correction for off-resonance effects and subject movement in diffusion MR imaging. NeuroImage 125:1063-1078

Jesper L.R. Andersson, Mark S. Graham, Eniko Zsoldos and Stamatios N. Sotiropoulos. 2016. Incorporating outlier detection and replacement into a non-parametric framework for movement and distortion correction of diffusion MR images. NeuroImage 141:556-572

Jesper L.R. Andersson, Mark S. Graham, Ivana Drobnjak, Hui Zhang, Nicola Filippini and Matteo Bastiani. 2017. Towards a comprehensive framework for movement and distortion correction of diffusion MR images: Within volume movement. NeuroImage 152:450-466

Jesper L.R. Andersson, Mark S. Graham, Ivana Drobnjak, Hui Zhang and Jon Campbell. 2018. Susceptibility-induced distortion that varies due to motion: Correction in diffusion MR without acquiring additional data. NeuroImage 171:277-295

Matteo Bastiani, Michiel Cottaar, Sean P. Fitzgibbon, Sana Suri, Fidel Alfaro-Almagro, Stamatios N. Sotiropoulos, Saad Jbabdi and Jesper L.R. Andersson. 2019. Automated quality control for within and between studies diffusion MRI data using a non-parametric framework for movement and distortion correction. NeuroImage 184:801-812

SQUAD: Group

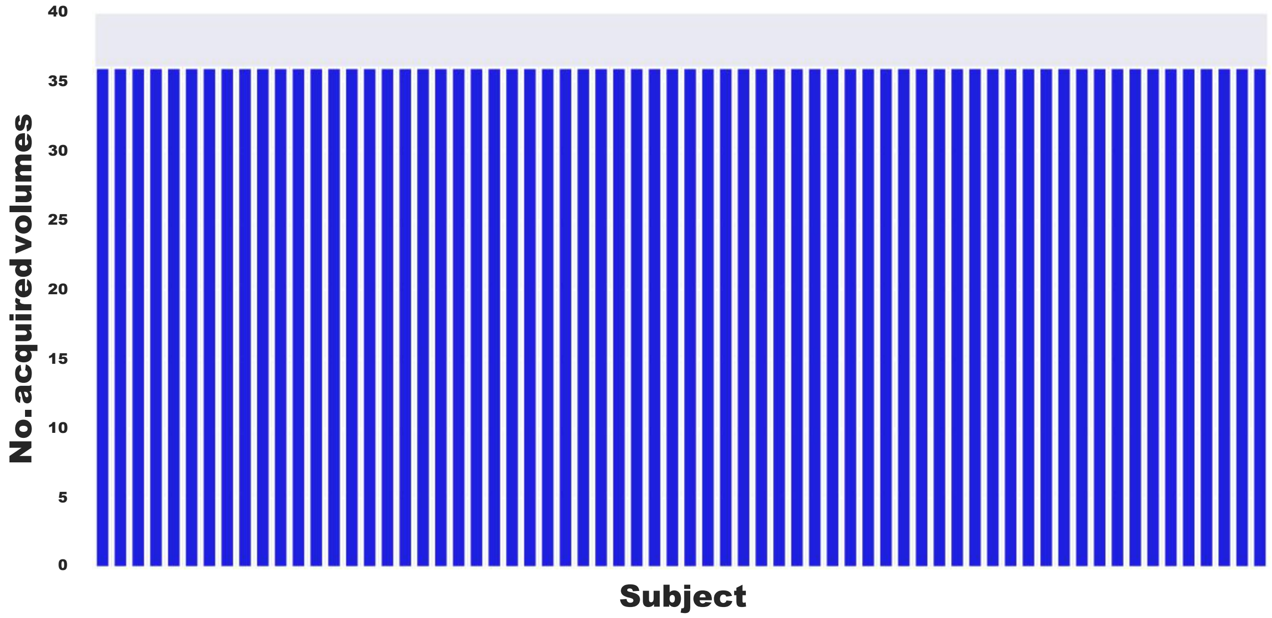
No. acquired volumes
40
35
30
25
20
15
10
5
0
Subject

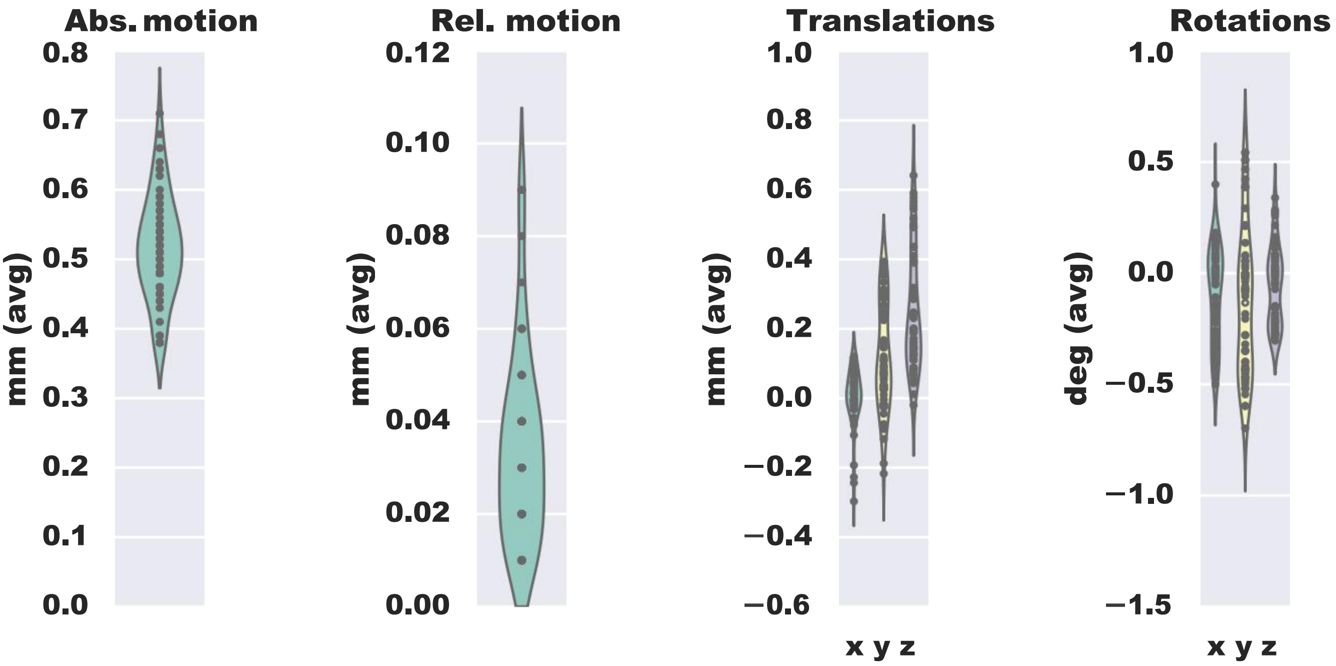
Abs. motion
mm (avg)
0.8
0.7
0.6
0.5
0.4
0.3
0.2
0.1
0.0
Rel. motion
mm (avg)
0.12
0.10
0.08
0.06
0.04
0.02
0.00
Translations
mm (avg)
1.0
0.8
0.6
0.4
0.2
0.0
−0.2
−0.4
−0.6
x y z
Rotations
deg (avg)
1.0
0.5
0.0
−0.5
−1.0
−1.5
x y z

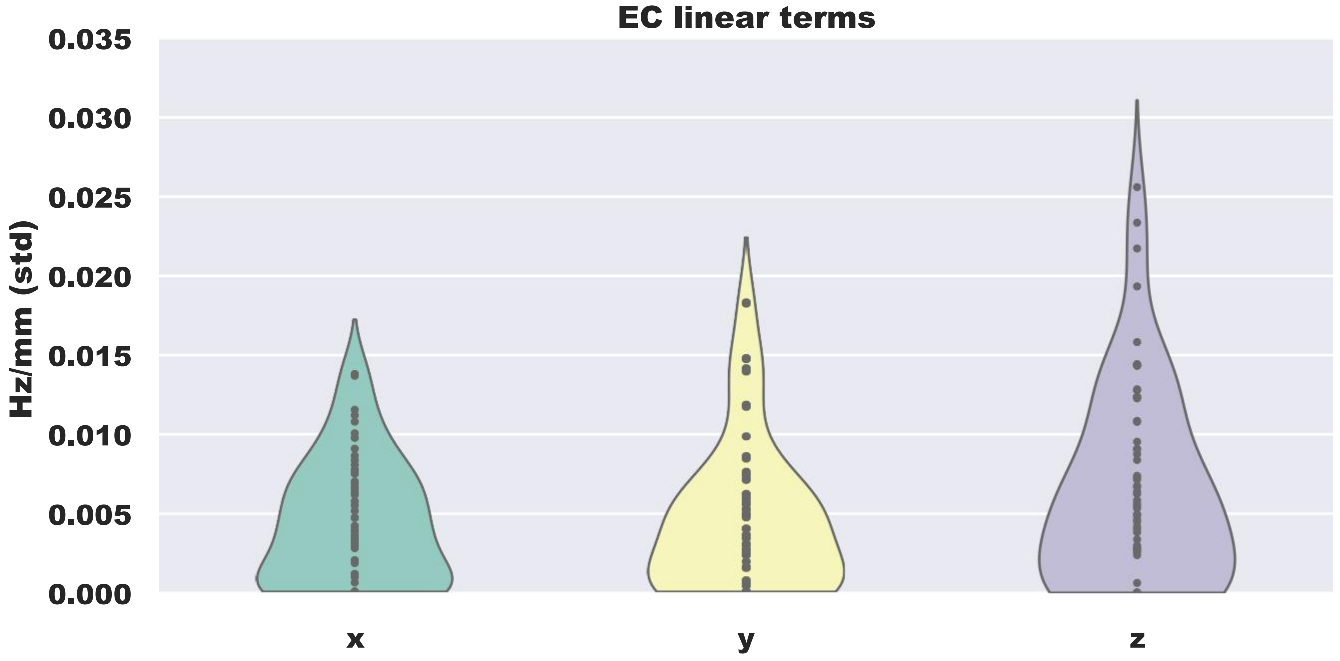
EC linear terms
Hz/mm (std)
0.035
0.030
0.025
0.020
0.015
0.010
0.005
0.000
x
y
z

SQUAD: Group

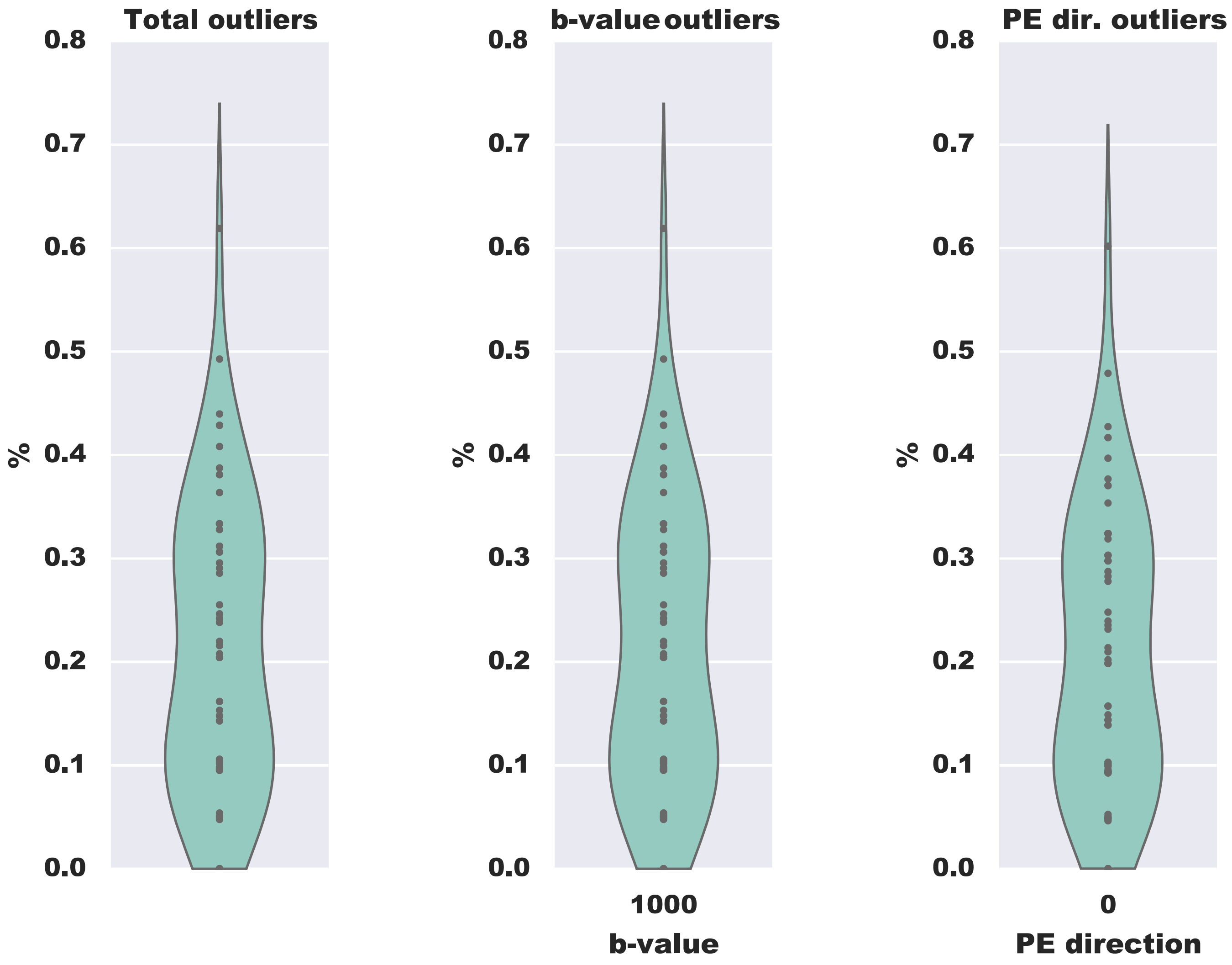
Total outliers
b-value outliers
PE dir. outliers
%
0.0
0.1
0.2
0.3
0.4
0.5
0.6
0.7
0.8
1000
b-value
0
PE direction
